# Opinions of Health Professionals on Tailoring Reproductive Health Services to the Needs of Adolescents

**DOI:** 10.1155/2018/1972941

**Published:** 2018-06-14

**Authors:** Ellen Abrafi Boamah-Kaali, Seyram Kaali, Grace Manu, Stephaney Gyaase, Elisha Adeniji, Seth Owusu-Agyei, Kwaku Poku Asante

**Affiliations:** ^1^Kintampo Health Research Centre, Ghana Health Service, Post Office Box 200, Kintampo, Brong Ahafo Region, Ghana; ^2^University of Health and Allied Sciences, Institute of Health Research, PMB 31, HO, Volta Region, Ghana

## Abstract

**Introduction:**

Tailoring sexual and reproductive health programs and services to the needs of adolescents will help adolescents make informed decisions and choices regarding their sexual and reproductive health.

**Objective:**

To assess the opinions of service providers on tailoring sexual and reproductive health services to the needs of adolescents.

**Method:**

A qualitative study using indepth interviews was held among eight decision-makers and service providers in two hospitals within the Kintampo North Municipality and Kintampo South District as well as the Municipal and District Health Directorates in Kintampo North and South between April and May 2011.

**Results:**

All respondents expressed the opinion that it is a good idea to tailor sexual and reproductive health services to the needs of adolescents. They admitted that very limited sexual and reproductive health programs targeting adolescent needs were available in the study area. Service providers also reported very low levels of health facilities use by adolescents for sexual and reproductive health information and services. Health professionals attributed the poor sexual and reproductive health services utilization by adolescents to stigma from the society and attitudes of service providers.

**Conclusion:**

There are no targeted sexual and reproductive health programmes and services for adolescents. Services providers indicated that it is important to tailor sexual and reproductive health services to the needs of adolescents to prevent stigma, unwanted pregnancy, abortion, and sexually transmitted infections.

## 1. Introduction

Adolescents face a variety of health and developmental challenges; prominent among them are teenage pregnancy and the acquisition of sexually transmitted infections. At such a young age, these can affect the quality of their health as they grow.

Every year around the world, about 16 million female adolescents aged 15-19 years and some one million girls below age 15 deliver babies [1]. Teenage pregnancy is an important public health and developmental concern given its direct impact on the adolescent mother, her child, and the family [2–4].

Adolescents are also at risk of sexually transmitted infections (STIs). They are the second most vulnerable group affected by sexually transmitted infections worldwide [1]. STI acquisition predisposes them to poor reproductive health outcomes. Unfortunately, only a few of them are able to access any form of affordable healthcare when infected [5].

Abstinence provides a sure way of preventing teen pregnancy and STIs. However, some 20-50% of adolescents are sexually active [6–8] and for those sexually active, there is a need to intervene by providing appropriate sex education and reproductive health services. These interventions will likely help them to make responsible reproductive health decisions to prevent early pregnancy and sexually transmitted infections [9–12].

To achieve this goal, tailored reproductive health services should be made available, accessible, responsive, and user friendly for adolescents. After 1994 International Conference on Population and Development, sexual and reproductive adolescence has been a global policy agenda, with response from many governments. However, in many African countries, services targeting the reproductive health needs of adolescents are limited or nonexistent [13–17]. Some service providers are hesitant to extend reproductive health service to adolescents [18–20], making the health system unfriendly for them [14, 21, 22]. Some adults and service providers are of the opinion that adolescents remain unsullied and morally upright, till they are both physically and emotionally developed to enter into marriage [21, 23]. In response to this call for moral propriety, adolescents face barriers in finding information and care regarding issues of sexual intercourse, STIs, pregnancy, contraception, among others [6, 22]. Hence only a few adolescents use reproductive health service [6, 24, 25]. Some adolescents actually refuse to utilize services in response to the negative attitudes from healthcare providers [26, 27]. The WHO recommends healthcare providers to be nonjudgmental and considerate in their dealings with adolescents [28] in contrast to fear-based, negative, or judgmental perspective on sexuality among some of them. However, current practices in most facilities reflect negative and judgmental attitudes from providers.

This manuscript presents the opinion of service providers on tailoring reproductive health services to the needs of adolescents in Kintampo, a rural Ghanaian community.

## 2. Methods

### 2.1. Study Design

A qualitative study to explore opinions on reproductive health services delivery to adolescents among decision-makers and service providers was carried out from April to May 2011. The study took place at the Kintampo North Municipal Hospital, Kintampo South District Hospital as well as the two health directorates in Kintampo North Municipality and South District.

### 2.2. Study Area Description

The Kintampo area is in the middle belt of Ghana, made up of predominantly rural communities. It has a resident population of about 151, 000 residents, with at least half made up of females. Subsistent farming is the most predominant occupation. Educational attainment is low for the general population. About 75% of adolescents are in school [29]. High levels of school dropout attributable to teenage pregnancy and marriage are common among the females. Two government owned hospitals, four private clinics, seven health centres, and about 27 functional community-based health planning and services (CHPS) facilities are located in the study area. Contraceptive use is low, with 25% among the general population [30] and 23% among adolescents [7].

### 2.3. Population

Health professionals made up of service providers and decision-makers in the health system formed the study population.

### 2.4. Sampling Procedure

Eight health professionals made up of 3 medical doctors, 2 public health nurses, and 3 midwives took part in the study. These were purposively selected from the Kintampo Municipal Hospital and Kintampo South District hospital and the Health Directorates in Kintampo North Municipality and Kintampo South District.

### 2.5. Instruments

A two-paged qualitative topic guide was developed for indepth interviews (IDIs) among the health professionals. This guide was pretested and the final version after revisions was used for data collection.

### 2.6. Data Collection Process

Indepth interviews among service providers were conducted face to face and facilitated by a 38-year-old female researcher with a bachelor's degree training in public health. She was assisted by a 37-year-old male note-taker with a bachelor's degree training in Sociology, who observed gestures in addition. Interviewers were trained for a day on study instruments and data collection processes. Prior to commencement of interviews, both the moderator and the note-taker introduced themselves to the respondents and established rapport with them. Reasons for the research were explained to participants and to allow them declare any conflicts of interest for the study being carried out. Interviews were guided by a pretested topic guide with open-ended questions. The moderator sought the consent of respondents to tape-record and take notes of all discussions, assuring respondents of confidentiality and anonymity. Interviews were done at health facilities in a private place convenient for participants. To ensure privacy, nonstudy participants were not allowed to be present. Data were collected to point of saturation. Each indepth interview lasted about forty minutes. There were no repeat interviews and no transcript was returned to participants for comment or clarification. All interviews were carried out in English language and audios were transcribed verbatim.

Data collection processes were supervised by a Research Fellow with a Master's degree in public health.

### 2.7. Data Management and Analysis

Qualitative data, involving IDIs among health professionals (service providers and decision-makers), were managed using QSR NVivo qualitative data management software (version 9; QSR International Pty Ltd, Doncaster, VIC Australia).

Recorded audios of IDIs were transcribed verbatim. Transcripts were checked for accuracy and completeness by listening to audios and matching them against the transcripts. Data was coded according to the emerging themes. Thematic analysis was done under themes that best addressed study's objectives. Responses that best describe the various themes were used as quotes to support the findings.

### 2.8. Ethics Approvals

Scientific approval for this study was received from the Kintampo Health Research Centre Scientific Review Committee. Ethical approval was also received from the Institutional Ethics Review Committee of the Kintampo Health Research Centre (FWA00011103).

Trained researchers experienced in asking sensitive questions conducted the interviews. Participants' privacy was respected during the data collection process. Written informed consent was obtained from study participants after the aim and objectives of the study had been explained to them. All participants received copies of the informed written consent forms bearing their signature or thumbprint and the signature of the researcher or a designated person. Respondents were free to decline to participate, refuse to answer questions they did not feel comfortable with, or withdraw from the study.

Confidentiality of participants was protected at all times. Respondents were identified using their study codes or designation. Electronic data have been stored on password protected computers accessible only to study investigators.

## 3. Results

### 3.1. Basic Sociodemographic Characteristics of Respondents

A total of eight participants were interviewed. Three (03) of them were male doctors. Among these three doctors, one was the director of one of the health directorates. The other two (02) were medical superintendents of the two hospitals. The five (05) other respondents were females. Two (02) of them were public health nurses and the other three were midwives. Their ages ranged between 38 and 56 years. All of them had more than ten years of working experience.

### 3.2. Availability of Sexual and Reproductive Health Programs for Adolescents in Kintampo

We found in our study that there were no tailored sexual and reproductive health programs and services just for adolescents. Some of the reasons accounting for this situation they said stemmed from the society's disapproval of discussing sex and related issues among adolescents. A midwife shared her opinion on this as follows:

We don't have. I think traditionally, I mean from our people, they think that people below the age of 18 years are not supposed to talk about sex. So even if you, establish a clinic and say adolescent reproductive center, people should come for counseling and family planning services, very few people will come there. As soon as they see a young girl of that age coming to you, then they will say that this is a bad work. Because of that we hardly establish such a unit. (A 50-year-old midwife)

However, some mentioned the existence of school health programs at which periods sex education is given.

OK. Especially at the schools we have programmes with them. We go and then give them talks. The senior high and the junior highs. (A 56-year-old public health nurse)

They also mentioned that though there are no specific programs and services for adolescents, yet, adolescents are given the priority when they come to the main stream hospital for services.

“OK, much as I know, we don't have a specific corner for the adolescents but priorities are given to them when they come to the antenatal clinic and then the family planning. So we don't normally mix them with the adults. As soon as they come, you see that we pick them, attend to them fast so that they can go” (IDI with a 35 years midwife)

### 3.3. Seeking of Information and Services on Sex and Reproduction among Adolescents

Respondents opined that adolescents were challenged in seeking for information and services regarding sex and other reproductive health services. They face barriers from service providers. Adolescents are considered as bad children when they go to the facilities for services. The quote below illustrates this opinion:

…..The young ones who do not want to have children can go and have family planning services and counselling. But, like I said, if children, I mean below the age of 15, 14, 16 go there, they are likely to be frowned upon. They are considered as bad children so because of that they don't even feel comfortable to go there. (A 35-year-old medical doctor)

Some adolescents also feel shy in coming to the facility for services. They would rather go to the homes of services providers or come to the facility after working hours to seek services. Some adolescents are also unable to disclose to the service providers exactly what their needs are. This was shared by a public health nurse as illustrated below.

I know antenatal they come but when they are coming, they feel shy. Then family planning too, they hide. Normally in the evenings, they go to our health workers for the services. Then STIs you know even we the adults we find it difficult to report, they will come to the clinic and tell you their lower abdomen is paining them. But they will not tell you they have some discharges, some offensives and all those things unless you probe. (A 49-year-old public health nurse)

Specifically, on provision of services on abortion and postabortion complications, respondents said that adolescent do not come to the facility for abortion services. They usually go for unsafe abortions and are brought to the facilities when there are complications. A midwife had this to share.

Abortion you know in Ghana it is not legalized. So they hide and then do it. They go in for the unsafe ones. Unless they have problems before they are rushed to the hospital. For them to walk to our facility that they want this thing I don't think. (A 42-year-old midwife)

### 3.4. Health Worker Perception on the Reasons for Adolescents Nonutilization of Reproductive Health Services

Healthcare workers blamed nonfacility (health service) utilization among adolescents on stigma from other people in the community as well as the attitudes of service providers. They also indicated shyness on the part of some adolescents. Additionally, some admitted that they are not well oriented and so they hesitate to provide these services.

I think it's because of stigmatization, they think people might criticize them for being young girls and patronizing reproductive health service at the health facility. So some of them feel shy, find it difficult coming to the health facility to get advice on reproductive health issues. (A 54-year-old medical doctor)

…unfortunately, even those of us working in the health, we are not well oriented so when a young girl come and is talking about, and trying to talk about sex, it's likely to be frown upon. They would say, you small girl and you want to know this thing you are spoilt. (A 42-year-old midwife)

### 3.5. Perceptions on Need to Tailor Sexual and Reproductive Health Services to the Needs of Adolescents

The health professionals perceived adolescents to be an important component of the society with special needs. They were optimistic that tailoring reproductive health services to their needs will reduce the rates of teenage pregnancy, abortion, STIs, and other complications. They shared these opinions.

I think it's important if we tailor services to the needs of adolescent to prevent stigmatization, unwanted pregnancies, abortions and HIV/AIDS. So I think it's a good idea and a good activity to tailor service specifically to adolescents. (IDI with a director of health services)

It's very necessary…, it's very necessary because they form a very important group of our society and they are many so their needs should be taken care of especially counselling services. Counselling and teaching them how to live responsible sexual life is very important because especially in this era of STI infections here and there, they need to be given the needed education and so it very important. (A 38-year-old medical doctor)

### 3.6. How to Tailor Sexual and Reproductive Health Services to Adolescent's Needs

Service providers and health managers indicated that creating dedicated space purposively for adolescents will boost their confidence to seek information and services and they had plans of pursuing this goal.

…we want to setup an adolescent friendly corner so that they can walk in. There, we have a lot of activities like showing films. There will be handouts, all sorts of magazines. It's complete…, we call it “adolescent clinic”, they come there for all sort of interactions with their peers and all those things. (A 49 year old public health nurse)

it's my vision to get a place for them where all the services that are required by them are met, so that at least they would also feel, they are being catered for. (A 56 year old public health nurse)

However, they said setting up a place like this would pose some challenges and they shared some of the anticipated challenges, including ways to surmount them.

### 3.7. Possible Challenges with Setting up Youth Friendly Centres

Respondents enumerated several foreseeable challenges of setting up youth friendly centres. They indicated that the sociocultural believes in Ghana do not permit premarital sex. Hence providing such facilities and services will meet some agitations from the wider community. It will be thought of as rather influencing adolescents to be sexually active. They also mentioned the unwelcoming attitudes of service providers as a big challenge because of their limited appreciation of adolescents' needs. Further, they mentioned inadequate staffing and lack of infrastructure. These themes have been illustrated with quotes as below.

### 3.8. Societal Disapproval

*“Oh! Maybe because of our socio-cultural beliefs and attitudes. The old people will think we are rather spoiling them. Like you know, they don't want us to talk about sex to these teenagers, they don't want us to talk about family planning to them. So they may think we are rather spoiling them instead of opening their minds on this sexual education and all those things.” (56-year-old public health nurse)*

### 3.9. Attitude of Service Providers

*I think the most important challenge is the attitude of the health staff towards the needs of this very people. We turn to underrate their needs. We think that they are young especially when they come with problem of sexual life. Abortion services, coming to get information about people and their sexual life, we turn to shun them thinking that they are spoiled because in those days' people were not talking about that. That is the important thing. The other thing is because of our attitude where even when they come with problems like STI's they are unable to express themselves because they would be considered very bad. So that is another challenge. (A director of health services)*

### 3.10. Inadequate Staff

*Well one of major problems is inadequate reproductive health staff. For example, in this particular hospital we have only four midwives. One comes in the morning, one in the afternoon, one is always on call or on leave or night rest after the duty. So when they (the adolescent) come and the midwives are very busy they don't seem to get the attention they need so it's one of the problems. (A 35-year-old medical doctor)*

### 3.11. Infrastructure

*I think infrastructure. Inadequate infrastructure. We have to get a specific place where they will get their privacy and all that. So I think that one too is not quite good (A 35-year-old midwife).*

### 3.12. Strategy to Surmount the Challenges

To surmount the challenges, participants mentioned the need to educate the general population and important stakeholders in the community.

OK. What I think we can do is we have to start with intensification on education. Start from our local people, in the communities, in the churches, radio talk shows so that we will just prepare their minds, sensitize them, let them know that if we expose these children to all these services, it will rather help. It will help a lot than leave them in dark then they will go and they will become casualties. (A 49-year old public health nurse)

...being a preventive nurse, I want to get on the field. I want to get the opinion leaders, the chiefs and then the assemblymen and then the CBS and the TBAs themselves. (A 56-year-old a public health nurse)

## 4. Discussion and Recommendation

Our study explored the opinions of services providers and decision-makers on tailoring reproductive health services to the needs of adolescents in the Kintampo area located in the middle belt of Ghana.

We found limited to nonexistent availability of reproductive health programs and services specifically targeting adolescent needs. Howbeit, adolescents need information for informed decision-making regarding their sexual and reproductive health [9, 10]. This can be through availability of tailored sex education programs coupled with appropriate service provision. There are economic and public health costs to not investing in adolescent sexual and reproductive health [31, 32]. Our study results are similar to that of Bankole et al., (2010) which found that access to sexual and reproductive health services including sexual education was still a challenge, worldwide [13]. A recent review of progress with adolescent sexual and reproductive health over the past twenty years showed a chronic lack of access to sexual and reproductive health information and services [15]. Some other studies have reported similar findings [14, 16, 17]. One prominent reason for the limited adolescent sexual and reproductive health programs in the study area was identified as society's disapproval of premarital sex and the introduction of adolescents to issues related to sex and sexuality. This reason has also been cited in other studies [23, 27]. Sex education and service provision to adolescents is believed to encourage adolescents to be sexually promiscuous [23, 33]. There is however no scientific evidence to back this claim. Studies have rather shown that adolescents are better able to make informed decisions about their reproductive health [11] and have better reproductive health outcomes when provided with the necessary education, information, and services [9, 12].

One of the main objectives of the Ghana Adolescent Reproductive Health policy of (2016) is to “promote programmes that will improve the knowledge of adolescents on sexual and reproductive health which will in turn guide them to develop socially acceptable and responsible attitudes towards sex and sexuality” [34]. The health system has a crucial role to play, in ensuring that such programs are designed for the youth especially for those in hard to reach areas, with support from other agencies like the Ministry of Education, Youth, and Social Welfare. It is also important that adolescents are involved in the design of context specific programs that target their needs to ensure comprehensiveness and also to ensure that adolescents feel involved. This practice has been found to be very helpful in program designs for adolescent [35].

Service providers also mentioned that only a few adolescents in the study area came to the facilities for sexual and other reproductive health services. This feedback reflects findings of studies from another study among young people aged 15 to 24 year in another rural Ghanaian setting. In that study, less than half of adolescents used reproductive health services [25]. Another study by Abajobir and Seme (2013) in rural Ethiopia found only one-fifth of adolescents using reproductive health services [24]. The low levels of service utilization among adolescents could be as a result of the limited access to facilities and services [13, 15, 16]. However, unwelcoming attitudes of the healthcare providers have been found to be a huge barrier for visiting health facilities for reproductive health services. In the study by Nalwadda and his colleagues in Uganda, some adolescents were actually refused reproductive health services by service providers [20]. Wood and Jewkes (2006) also found in their study that adolescents were embarrassed and scolded by nurses for seeking reproductive health services [22]. These health worker attitudes made it hard for adolescents to conveniently walk into health facilities to access SRH services. In Ahanou's study, some service providers insisted that adolescents should not be provided with contraceptive services [21]. Similar findings have been reported in some other studies [14, 18–20, 23]. It is not surprising therefore when service providers in our study attributed reasons for nonservice utilization among adolescents to include embarrassment, stigmatization, and scolding from service providers and the society.

Some health providers and decision-makers however acknowledged the need to tailor reproductive health service to adolescent needs. They showed enthusiasm and commitment to initiate programs and services specific to adolescents' needs. However, they envisaged challenges from different directions including societal disapproval, poor health worker attitudes, and inadequate staffing and infrastructure. The society's disapproval of provision of sexual and reproductive health services to adolescents is deeply rooted in our religious and cultural beliefs relating to morality [36]. Religion and culture form the core fabric of people's belief systems [37]. Changing the societal perspectives on issues of morality especially relating to providing sexual and reproductive health services requires high level of commitment, hard work, and proven evidence of what works [38]. Some decision-makers in the health system mentioned the need to educate the general public including religious leaders and traditional rulers on the need to make sexual and reproductive health information and services available to young people. This is a huge venture that requires hard work and commitment [38]. However, with continued education and advocacy, there can be a change in relation to poor health worker attitudes; service providers are part of the society and share in the believes of the society. Some are unable to separate their personal believes on morality from the ethics of their job [39]. With training and reorientation, it is possible to bring such service providers on board, to provide to the best of their knowledge and training, information, and services needed by adolescents to improve their sexual and reproductive health [23]. On the issue of inadequate staff and infrastructure, where there is a will, there is always a way. For a start, service providers can double task as the case has always been. Health facilities can designate a single room just for adolescents to conveniently walk in for services. Also, the Ministry of Health can partner with cooperate entities and nongovernmental organisations working in the area of ASRH, to support with infrastructure in the long term.

## 5. Conclusion

There are no targeted sexual and reproductive health programmes and services for adolescents. Important stakeholders in the reproductive health service provision and decision-making acknowledge the need to tailor reproductive health services to the needs of adolescents. They plan to pursue this agenda through the education of the general population, opinion leaders, and all other stake holders, through divers' media. The next important step they indicated is to establish adolescent friendly corners where adolescents can conveniently seek information and everything related to their sexual and reproductive health.

## Data Availability

Data and other study materials are available on request. Interested persons may contact the corresponding author.
